# Performance of five pulse oximeters to detect hypoxaemia as an indicator of severe illness in children under five by frontline health workers in low resource settings – A prospective, multicentre, single-blinded, trial in Cambodia, Ethiopia, South Sudan, and Uganda

**DOI:** 10.1016/j.eclinm.2021.101040

**Published:** 2021-07-22

**Authors:** Kevin Baker, Max Petzold, Akasiima Mucunguzi, Alexandra Wharton-Smith, Emily Dantzer, Tedila Habte, Lena Matata, Diana Nanyumba, Morris Okwir, Monica Posada, Anteneh Sebsibe, Jill Nicholson, Madeleine Marasciulo, Rasa Izadnegahdar, Tobias Alfvén, Karin Källander

**Affiliations:** a Malaria Consortium, London, United Kingdom; b Department of Global Public Health, Karolinska Institutet, Stockholm, Sweden; c School of Public Health and Community Medicine, Institute of Medicine, University of Gothenburg, Gothenburg, Sweden; d University of the Witwatersrand, Johannesburg, South Africa; e Malaria Consortium, Kampala, Uganda; f Malaria Consortium, Phnom Penh, Cambodia; g Malaria Consortium, Addis Ababa, Ethiopia; h Malaria Consortium, Juba, South Sudan; i Malaria Consortium, Raleigh, United States; j Bill & Melinda Gates Foundation, Seattle, United States; k Sachs’ Children and Youth Hospital, Stockholm, Sweden; l Programme Division, Health Section, UNICEF, New York, United States

**Keywords:** Childhood pneumonia, Severe illness, Low-income country, Diagnostic aids, Pulse oximeter, Health worker performance

## Abstract

**Background:**

Low blood oxygen saturation (SpO_2_), or hypoxaemia, is an indicator of severe illness in children. Pulse oximetry is a globally accepted, non-invasive method to identify hypoxaemia, but rarely available outside higher-level facilities in resource-constrained countries. This study aims to evaluate the performance of different types of pulse oximeters amongst frontline health workers in Cambodia, Ethiopia, South Sudan, and Uganda.

**Methods:**

Five pulse oximeters (POx) which passed laboratory testing, out of an initial 32 potential pulse oximeters, were evaluated by frontline health workers for performance, defined as agreement between the SpO_2_ measurements of the test device and the reference standard. The study protocol is registered with the Australia New Zealand Clinical Trials Registry (Ref: ACTRrn12615000348550).

**Findings:**

Two finger-tip pulse oximeters (Contec and Devon), two handheld pulse oximeters (Lifebox and Utech), and one phone pulse oximeter (Masimo) passed the laboratory testing. They were evaluated for performance on 1,313 children under five years old by 207 frontline health workers between February and May 2015. Phone and handheld pulse oximeters had greater overall agreement with the reference standard (56%; 95% CI 0.52 - 0.60 to 68%; 95% CI 0.65 - 0.71) than the finger-tip POx (31%; 95% CI 0.26 to 0.36 and 47%; 95% CI 0.42 to 0.52). Fingertip POx performance was substantially lower in the 0–2 month olds; having just 17% and 25% agreement. The finger-tip devices more often underreported SpO_2_ readings (mean difference -7.9%; 95%CI -8.6,-7.2 and -3.9%; 95%CI -4.4,-3.4), and therefore over diagnosed hypoxaemia in the children assessed.

**Interpretation:**

While the Masimo phone pulse oximeter performed best, all handheld POx with age-specific probes performed well in the hands of frontline health workers, further highlighting their suitability as a screening tool of severe illness. The poor performance of the fingertip POx suggests they should not be used in children under five by frontline health workers. It is essential that POx are performance tested on children in routine settings (in vivo), not only in laboratories or controlled settings (in vitro), before being introduced at scale.

**Funding:**

Bill & Melinda Gates Foundation [OPP1054367].

**Research in context**

**Evidence before this study**

We searched PubMed, the Cochrane Controlled Clinical Trials Register, and ClinicalTrials.gov database for relevant published articles and current trials assessing the accuracy, usability and acceptability of pneumonia diagnostic aids for use by frontline health workers in children under five. We used the search terms “ pulse oximeter” or “severe pneumonia illness screening aid” or “severe illness diagnostic device” or “diagnostic” and “community health worker” or “frontline health worker” and “children under five” and “clinical trial” or “randomised control trial” or “study”. We limited the search to studies published from Jan 1, 1990 to Jan 1, 2018. We found no Cochrane systemic reviews or large-scale randomised control trials of pulse oximeters. We found a number of small-scale studies of various pneumonia diagnostics aids for frontline health workers, but nothing specifically for pulse oximeters used at community level in these settings.

**Added value of this study**

To the best of our knowledge, our study is the first large, multi-centre trial evaluating the use of pulse oximeters as severe illness diagnostic screening aids by frontline health workers in children under five. Our study, with its pragmatic design for resource poor settings, makes the results generalisable to other similar settings and populations.

**Implications of all the available evidence**

We saw varied agreement between the five pulse oximeters tested and the reference standard; the findings of our study showed that multi-probe phone or handheld devices are required for effective screening of children under five for hypoxaemia as an indicator of severe illness. Furthermore, our study highlighted that it is essential that devices also are tested on a wide age range of children in routine settings by a representative sample of health workers to fully understand their performance.

Alt-text: Unlabelled box

## 1 Introduction

Low blood oxygen saturation, or hypoxaemia, is an indicator of severe illness including pneumonia and sepsis, and has been identified as a predictor for morbidity and mortality in children with respiratory illness [1,2]. However, hypoxaemia is poorly identified based on clinical findings alone [3], and the inability of health care workers to promptly detect and refer these children, whose lives are in danger, leads to the death of many children [4]. While pulse oximetry is a reliable and non-invasive method for identifying children with hypoxaemia, through measuring non-invasive peripheral oxygen saturation (SpO_2_), pulse oximeters are rarely available outside higher-level facilities in resource-constrained countries, due to cost implications, plus a lack of perceived need by policy makers and health workers [5]. The current 2014 World Health Organization (WHO) Integrated Management of Childhood Illness (IMCI) chart booklet includes pulse oximetry as optional rather than mandatory, and stipulates a threshold of <90% to indicate hypoxaemia requiring immediate referral to hospital.

More recently, and partly in response to the increased focus on the importance of better access to pulse oximeters and oxygen therapy in sub-Saharan Africa and Southeast Asia, new pulse oximeters have been developed by industry, academia and other partners to improve the accuracy and effectiveness of detecting this indicator of severe illness in resource-poor contexts, leading to higher referral rates and ultimately better health outcomes [6], [7], [8], [9], [10], [11]. Recent feasibility studies have shown that health workers in these settings can use these devices effectively [3,12], but usage still remains low [13,14].

This is the first large-scale study, which aimed to identify the most accurate, acceptable, scalable and user-friendly pulse oximeters for the detection of hypoxaemia in children by community health workers (CHWs) and first level health facility workers (FLHFWs) in four countries: Cambodia, Ethiopia, South Sudan, and Uganda.

## 2 Methods

### 2.1 Study design

A multi-centred, prospective, single-blinded, comparison of performance of devices to detect hypoxaemia in the hands of CHWs and FLHFWs was conducted in Cambodia, Ethiopia, South Sudan and Uganda. Detailed description of the methods are published elsehwere [19]. Study sites of district hospitals were selected in each of the four countries after confirming that they had CHWs who were actively delivering iCCM services. The hospitals were chosen based on an analysis of the patient flow that could generate the required sample size. These district hospitals were not the typical workplaces for the CHWs or FLHFWs, who were brought there for up to five days to conduct the evaluations. The research sites were: Borkeo Hospital in Banlung town, Ratanakiri province in the north of Cambodia; Yergalem District Hospital in Yergalem town, Sidama Zone, Southern Nations and Nationalities and People's Region (SNNPR) in South-western Ethiopia; Aweil General Hospital in Aweil town, Northern Bahr el Ghazal state, in the north of South Sudan and Mpigi Health Centre IV in Mpigi district, in central Uganda. All sites were equipped with functioning oxygen management capabilities and the research teams ensured that first line antibiotic treatment for pneumonia was available. All research sites were below 2000 metres elevation (which would change the definition/threshold of hypoxaemia), with Yergalem having the highest elevation at 1776 metres.

### 2.2 Participants

All children aged 2–59 months who sought care at these facilities, presenting with cough or difficulty in breathing and all young infants aged zero to less than two months were asked to participate. Children were excluded if they presented with an illness of greater than two weeks duration; were assessed as having one or more danger signs (severe dehydration, agitation, inconsolable, neck stiffness, active convulsions/fits, unconscious/lethargic, not breastfeeding and vomiting everything); had a caregiver who were less than 18 years of age or who did not give consent; were in an in-patient facility with severe burns; or if the child/young infant was not eligible for research procedures as advised by the supervising clinician was also excluded from the study.

### 2.3 Procedures

#### 2.3.1 Device identification process

The device identification and testing processes consisted a number of distinct phases. Firstly, a landscape review was conducted to document existing pulse oximeters that could potentially be used by CHWs [15]. Formative research, including focus group discussions with CHWs, was undertaken to document current practices and to inform the attributes used in subsequent device scoring. Consultations were also held with key Ministry of Health (MoH) personnel in each country to assess device acceptability and scalability criteria using pile sorting methodology. The results of the formative research was published elsewhere [16].

The 32 devices identified in the landscape review were scored and ranked using 20 device attributes including measures of usability, utility, scalability and user acceptance (appendix 1) [17]. Of these, the top seven scoring pulse oximeters were selected for laboratory testing by the project scientific advisory committee, composed of 12 global experts in child health. Of the seven selected POx, four were fingertip devices, two were handheld and one was a mobile phone with a SpO2 application installed and with external probes. Five samples of each seven devices were tested for accuracy and environmental robustness at the TUV Rheinland Laboratory in Budapest, Hungary in December 2014; a laboratory with previously documented experience in evaluating medical devices. All tests were based on ISO standard 80,601–2–61 for pulse oximeters. After being exposed to the environmental robustness tests each device was subjected to accuracy testing on a Fluke SPOT Light pulse oximeter tester (Fluke Biomedical, Everett, WA, USA) [18], which provided a simulated oxygen saturation at eight different saturation points between 80% to 100%. The performance time, i.e. the time in seconds from initiation to the recording of an acceptable reading, and the lag time, i.e. the time in seconds from sensor placement to acceptable reading being recorded, were documented. Each set of tests was conducted ten times to calculate an average value for each device tested. The accuracy was calculated using the maximum allowable tolerance value for SpO2 (±2%) between the saturation value on the simulator and the displayed value on the device being tested. Testing also included cyclical heat testing (72 hour cycle ranging from −20° to +40 °Celsius); damp heat testing (50 °Celsius with 85% humidity over 72 h); dry heat testing (60 °Celsius over 72 h); vibration and shock testing (10 Hz to 1000 Hz: 1,0 (m/sq) sp/Hz for 30 min); free fall testing (ten drops: five to front side and five to back side from a height of one metre onto concrete); and dust testing (devices placed in dust shed for eight hours (IP5X test)). Two pulse oximeters did not pass all the laboratory tests (one failing the performance test on simulators and one failing the dust test) and hence five devices were taken forward for performance testing by frontline health workers Table 1 [17].

**Table 1:** Description of the test devices used in the performance evaluation.

**Contec fingertip paediatric pulse oximeter (Model: CMS50QB)** Measures oxygen saturation and pulse rate through attaching the device to the patient's finger or toe. Comes with two rechargeable batteries which can be used up to 300 times between charging. Recommended for use with paediatric patients due to its smaller size. CE approved as a class IIb medical device. Dimensions: 46 mm x 40 mm x 29 mm. Weight: 35 g. Cost: Approx. $40.	
**Devon fingertip pulse oximeter (Model: PC600)** Measures oxygen saturation and pulse rate through attaching the device to the patient's finger or toe. Comes with a rechargeable battery and recommended for use with paediatric patients due to its size. Device has programmable audible alarms. CE approved as a class IIb medical device. Dimensions: 55 mm x 40 mm x 30 mm. Weight: 80 g. Cost: Approx. $80.	
**Lifebox handheld pulse oximeter (Model: AH-M1)** Measures oxygen saturation and pulse rate through attaching the probe to the patient's finger or toe. Can be used on adults, paediatric and neonatal patients. Supplied with reusable adult, neonatal and paediatric probes. Device has visual and audible alarms. Powered by both battery and mains and is supplied with a rechargeable lithium battery. CE approved as a class IIb medical device. Dimensions: 58.5 mm x 123 mm x 28 mm. Weight: 200 g (without batteries). Cost: approx. $250.	
**Utech handheld pulse oximeter (Model: UT100)** Measures oxygen saturation and pulse rate through attaching the probe to the patient's finger or toe. Can be used on adults, paediatric and neonatal patients. Supplied with an adult reusable probe as standard, but paediatric and neonatal reusable probes are available. Device has visual and audible alarms. Operated with four AA batteries but can be used with rechargeable batteries (purchased separately). CE approved as a class IIb medical device. Dimensions: 75 mm x 135 mm x 28 mm. Weight: 158 g (without batteries). Cost: approx. $100.	
**Masimo handheld/mobile phone pulse oximeter (Model: iSpO2 Rx)** Measures oxygen saturation, respiratory rate and pulse rate through attaching the probe to the patient's finger or toe. Can be used on adults, paediatric and neonatal patients. Supplied with single and multi-use adult, paediatric and neonatal probes. The results are displayed on a connected Android phone or iPhone. The device features low profusion and motion software supporting SpO2 assessments. CE approved as a class IIb medical device. The device is charged through the mobile phone and does not require an independent power source. Dimensions: 25 mm x 10 mm x 5 mm. Weight: 30 g (without phone). Cost: approx. $250 (without phone).	

The research team recruited children in the waiting rooms of the outpatient departments of the participating hospitals. Each child had a pair of device tests on them. To ensure parity of testing, the sequence of use of test devices was randomised (using https://www.random.org/) per study visit day, ensuring an even distribution of devices tested. Data on SpO_2_ measurements of the test device, along with simultaneous reference standard readings, were recorded on paper forms. The reference standard, the Masimo Root patient monitor and connectivity platform with Radical 7 pulse oximeter with paediatric and neonatal probes [20], is an automated continuous monitor that was connected to the child's left or right index finger, and provided a simultaneous SpO_2_ measure as the test device used by the CHW/FLHFW. All pulse oximeter devices tested used the recommended sensor-to-infant-first (STIF) technique, where the sensor is first placed on the child before the pulse oximeter is switched on [21]. The CHW/FLHFW was asked to classify the test device SpO_2_ reading into “hypoxemic” or “not hypoxemic” using the WHO recommended cut-off at < 90%, and their classification was recorded. In addition, performance and lag time to obtain a reading for each of the test devices were documented. After all the field work had been completed, a sample of one device per country was sent for a second set of performance and accuracy tests in the laboratory using a Fluke simulator.

#### 2.3.2 Ethics

The study was approved by ethical review boards in each study country at national or regional level - in Cambodia from the National Ethics Committee for Health Research (Ref: 0146 NECHR), in Ethiopia from the Southern Nations Nationalities Peoples' Region Health Bureau Health Research Review Committee (Ref: 6–19/10,342); in South Sudan from the Research and Ethics Committee at the Government of South Sudan, Ministry of Health (Dated 23/05/2014); in Uganda, from the Uganda National Council for Science and Technology (UNCST) (ref. HS 1585); and by the Regional Ethics Committee in Stockholm, Sweden (Ref. 2017/4:10). The study protocol is published [19] and is registered with the Australia New Zealand Clinical Trials Registry (ANZCTR) (Ref: ACTRrn12615000348550). All participants and accompanying caregivers provided written consent before being enroled in the study.

#### 2.3.3 Outcomes

The primary outcome was agreement between each CHW/FLFHW measurement with the test devices and that of the reference standard, calculated as the proportion of the SpO_2_ measurements for each of the test devices that were within +/- 2% of the reference standard. Secondary outcomes included the agreement in classification of the SpO_2_ rate into hypoxaemia (<90%) or non-hypoxaemia obtained by the CHW/FLHFW and the reference standard. It was measured using the positive and negative percent agreement (PPA and NPA) for each device, as well as Cohen's Kappa statistic (κ), the mean (SD) performance time to obtain a reading for each of the selected devices, the mean (SD) lag time to obtain a reading for each of the selected devices, and the percent of attempts that resulted in a reading failure, classified as three unsuccessful attempts at getting a SpO_2_ reading.

## 3 Statistical analysis

For descriptive statistics, frequencies and proportions were calculated for categorical data whereas mean, standard deviation, median, minimum and maximum were calculated for count data. The unit of analysis for both the primary and secondary outcomes was the device measurement and analysis were done per-protocol-analysis, excluding children who were not calm at the time of measurement. The sample size for this study was based on the calculation used in an earlier reported respiratory rate (RR) timer evaluation [22,23]. Based on the primary outcome, calculated as the proportion of the SpO_2_ measurements for each of the test devices that were within +/- 2% of the reference standard, with a 50% level of agreement and a 95% CI ±2% SpO_2_ we further calculated we would need a sample size of 384 device measurements per strata, requiring 1536 device measurements for each device in the study overall. In addition, PPA and NPA were calculated using the CHW/FLHFW classification of the SpO_2_ rate of the test device compared to the classification of the reference standard measurements. The Kappa (κ) statistic was used to calculate the agreement between the classification of the SpO_2_ rates (normal or hypoxemic) measured by the CHW/FLHFW and reference standard for each device and age group, and is expressed as a fraction of the maximum difference [24]. κ ranges from −1 to 1 and is a useful measure to demonstrate performance in the absence of a gold standard. When interpreting κ values Altman considers agreement at <0·20 as poor, 0·21–0·40 as fair, 0·41–0·60 as moderate, 0·61–0·80 as good, and 0·81–1 very good [24]. Primary and secondary outcomes were calculated separately for each user group (CHW/FLHFW) and per device. Performance time (PT) and lag time (LT) for each device tested was recorded in seconds for each observation. In each country data was double entered in EpiData (www.epidata.dk). All analysis was done in STATA 13 (STATA Corp, College Station, TX, USA). The manuscript adheres strictly to the CONSORT guidelines for reporting trials.

### 3.1 Role of the funding source

The funder of the study had a role in the study conceptualisation and design, but not in the study site selection or data analysis. The corresponding author had full access to all study data and had the final responsibility for the decision to submit for publication.

### 3.2 Results

Of the seven devices tested in the laboratory, all were within ±2% of the simulator reading and therefore passed the accuracy testing stage. Out of the seven pulse oximeters, one fingertip device which did not provide an initial oxygen saturation reading, thus was not subjected to the other tests, one passed all of the tests except the dust exposure and the remaining five passed all of the tests and performed quite similarly.

A total of 207 CHWs and FLHFWs were trained in the study procedures. All passed the post-training competency test and participated in the device evaluation from February to May 2015. Overall, 43% were male, the mean age was 32·5 years, the mean time they had worked in their roles as CHWs or FLHFWs was 6·6 years and 13% had no formal education, with 21% having completed primary education, 31% secondary education and 35% having completed tertiary education.

**Fig. 1:**
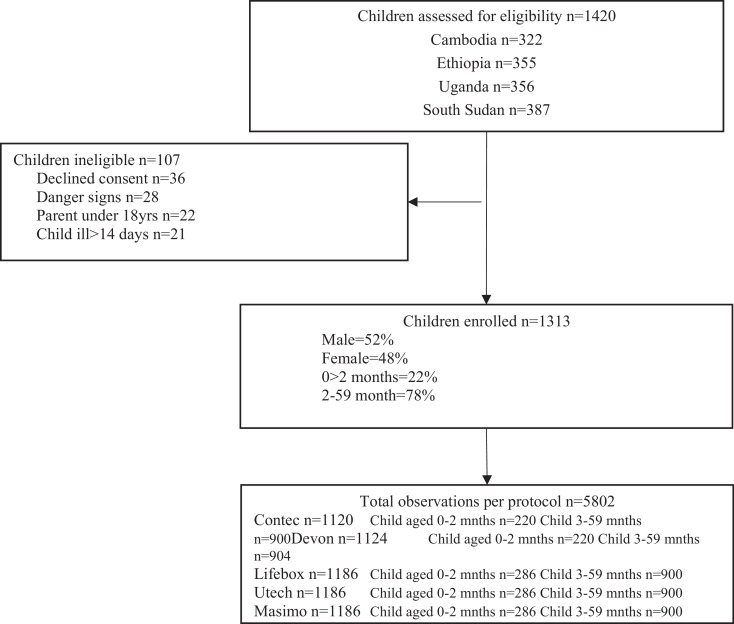
Trial profile for pulse oximeter performance evaluation.

A total of 1420 children were assessed for eligibility and 1313 were enroled in the study. The total number of observations from these children was 5802, with slightly less observations recorded in Cambodia compared to the other three research locations. Just over half (52%) of children were boys and 22% were less than 2 months of age. The mean age of all caregivers was 26·8 years (Figure 1).

While it was a requirement that all children were calm before the assessments, the research assistants also recorded the state of the child during assessments. The majority of children were calm (79%), while 10% were moving or agitated during the assessment and the remaining 11% did not have the state recorded. As the proportion of children who moved or were agitated was low, the following results were analysed per-protocol rather than intention-to-treat analysis, which would have excluded those who were moving or agitated. Out of all SpO_2_ measurements completed with the five test devices, a failure rate of 4·5% was recorded. The Utech handheld pulse oximeter had the highest failure rate (9%), followed by Contec (6%) and Devon (4%). Lifebox (2%) and Masimo (1%). No adverse events were recorded.

The primary outcome showed that the Masimo iSpO_2_ Rx handheld/phone pulse oximeter had the greatest agreement with the reference standard, with 68% of observations being within ±2% (Table 2). The handheld pulse oximeters (Lifebox and Utech) had similar levels of agreement (58% and 56%) whereas the fingertip pulse oximeters (Contec and Devon) had less agreement with the reference standard (47% and 31%). The level of agreement was generally lower in the younger children, with agreement in 51% and 34% with the handheld devices and 25% and 17% of observations with fingertip devices. Only the Masimo phone pulse oximeter had higher agreement in the younger children (72%) than in the older children (67%). While all devices under estimated SpO_2_ readings compared to the reference standard, the finger-tip devices were particularly more likely to under estimate SpO_2_.

**Table 2:** Performance of pulse oximeters, by device, when compared to the reference standard (Masimo Radical 7).

	**Contec (Fingertip)**	**Devon (Fingertip)**	**Lifebox (Handheld)**	**Utech (Handheld)**	**Masimo (Phone)**
**Primary outcome**					
±2 SpO2% Agreement Overall% n/N (95% CI)	31 303/1120 (26% - 36%)	47 454/1124 (42% - 52%)	56 609/1186 (52% - 60%)	58 596/1186 (54% - 62%)	68 784/1186 (65% - 71%)
Children aged 0 to 2 months% n/N	17 29/171	25 41/163	53 111/211	40 81/202	72 195/270
Children aged 2 to 59 months% n/N	34 274/817	51 413/812	57 498/869	63 515/821	67 589/879
**Secondary outcomes**					
Mean difference or ‘bias’ (95% CI)	−7.9 (−8.6 to −7.2)	−3.9 (−4.4 to −3.4)	−2.7 (−3.0 to −2.3)	−3.0 (−3.4 to −2.6)	−0.6 (−0.9 to −0.4)
Kappa value (κ) (95% CI), for agreement of classification of SpO_2_ as normal or hypoxaemia	0·19 (0·15 to 0·23) *n* = 1120	0·38 (0·34 to 0·44) *n* = 1124	0·48 (0·42 to 0.54) *n* = 1186	0·46 (0·40 to 0.52) *n* = 1186	0·67 (0·61 to 0.73) *n* = 1186
Children aged 0 to 2 months Kappa value (κ) (95% CI)	0·01 (−0·01 to 0·03) *n* = 220	0·00 (0·0 to 0·0)) *n* = 220	0·31 (0·21 to 0·41) *n* = 286	0·2 (0·01 to 0·30) *n* = 286	0·5 (0·38 to 0·62) *n* = 286
Children aged 2 to 59 months Kappa value (κ) (95% CI)	0·25 (0·19 to 0·31) *n* = 900	0·48 (0·42 to 0·54) *n* = 904	0·51 (0·45 to 0·57) *n* = 900	0·57 (0·51 to 0·63) *n* = 900	0·71 (0·65 to 0·77) *n* = 900
Positive percent agreement (PPA) Overall% (95%CI)	89 (80·0 to 94·8)	83 (72·4 to 90·1)	87 (77·6 to 92·8)	88 (79·4 to 94·2)	84 (74·5 to 90·0)
Children aged 0 to 2 months% (95%CI)	0 (0)	0 (0)	80·0 (28·4 to 99·5)	57·1 (79·4 to 94·2)	57.1 (74·5 to 90·0)
Children aged 2 to 59 months% (95%CI)	94·1 (80·3 to 99·3)	80·6 (64 to 91·8)	91·4 (76·9 to 96·8)	88·6 (73·3 to 96·8)	85·7 (69·7 to 95·2)
Negative percent agreement (NPA) Overall% (95% CI)	63·5 (60·3 to 66·6)	83·6 (81·0 to 85·9)	88·2 (85·7 to 89·9)	86·7 (84·3 to 88·8)	95·5 (94·1 to 96·6)
Children aged 0 to 2 months% (95%CI)	0 (0)	0 (0)	96·3 (91·5 to 98·8)	89·7 (83·0 to 94·4)	99·3 (96·0 to 100·0)
Children aged 2 to 59 months% (95%CI)	79·3 (75·1 to 83·1)	95·4 (92·9 to 97·2)	92·9 (90·0 to 95·2)	95·6 (93·1 to 97·4)	97·3 (65·3 to 98·7)
Mean performance time Seconds (SD)	25·6 (17·1)	26·1 (16·6)	47·6 (33·6))	60·9 (45·6)	57·5 (37·5)
Mean lag time Seconds (SD)	12·5 (8·8)	15·8 (10·1)	22·4 (13·6)	27·3 (13·6)	27·3 (13·9)

Using the standard WHO thresholds to classify the oxygen saturation into hypoxaemia or non-hypoxaemia, there was more variability in the secondary outcomes, especially in the agreement between the test devices and the reference standard in the younger children when compared to the older children. The Masimo phone pulse oximeter had the best agreement of all five pulse oximeters (κ=0·67) with moderate agreement (κ=0·5) for the younger children and good agreement (κ=0·71) for the older children. The Contec fingertip pulse oximeter had the poorest agreement with the reference standard across both age groups (κ=0·01– to 0·25).

There was no significant difference between the performance and lag time of the different test devices in each type of pulse oximeter tested and all were within the acceptable 60 second limit expected from pulse oximeters. After the field evaluation, a functioning sample of one device per country where again tested in the laboratory and all were functioning well. All were within ±2% of the oxygen saturation (SpO2) given by the simulator and therefore passed the post-field accuracy testing. The performance and lag time of all the devices were also within the acceptable range of 60 s. There was no significant difference in performance across the four countries where the performance evaluation was conducted.

FLHFWs using the test devices consistently had better agreement with the reference standard compared to the CHWs (Table 3). In the older children, the handheld pulse oximeters in the hands of FLHFWs had good agreement with the reference standard (κ=0·61–0·71), while they only had moderate agreement when used by CHWs (κ=0·47–0·60). FLHFW also performed better than the CHWs for both age groups when using the Masimo phone pulse oximeter.

**Table 3:** Kappa value (κ) for agreement of classification of SpO_2_ as normal or hypoxaemia between the two health worker types and Masimo Radical 7 reference standard, by device and age group.

	Age 0 to <2 months κ (95%CI)		Age 2–59 months κ (95%CI)		Overall κ (95%CI)	
Device	**CHW**	**FLHFW**	**CHW**	**FLHFW**	**CHW**	**FLHFW**
Contec	0·00** (0·00 to 0·00)	0·05 ** (−0·05 to 0·06)	0·21 (0·15 to 0·27)	0·39 (0·0.27 to 0.51)	0·15 (0·11 to 0·19)	0·32 (0·22 to 0.51)
Devon	0·00 ** (0·00 to 0·00)	0·00 ** (0·00 to 0·00)	0·42 (0·34 to 0·50)	0·67 (0·53 to 0.81)	0·32 (0·26 to 0.39)	0·55 (0·43 to 0·67)
Lifebox	0·12 (0·04 to 0·2)	0·59 (0·32 to 0·86)	0·47 (0·39 to 0·55)	0·61 (0·47 to 0·75)	0·42 (0·36 to 0.48)	0·61 (0·49 to 0·73)
Utech	0·10 (0·02 to 0·18)	0·53 (0·26 to 0·80)	0·51 (0·45 to 0·57)	0·71 (0·57 to 0·85)	0·39 (0·33 to 0·45)	0·67 (0·55 to 0·76)
Masimo	0·25 (0·13 to 0·18)	0·91 (0·64 to 1·18)	0·65 (0·574 to 0·72)	0·84 (0·70 to 0·98)	0·59 (0·53 to 0.65)	0·86 (0·72 to 0.99)

**small sample.

## 4 Discussion

It has been suggested that the inclusion of pulse oximetry for the management of severe illness in resource poor settings can improve health outcomes [12,25]. In 2014 the WHO amended their guidelines on facility based management of pneumonia to include the use of pulse oximetry, when these devices are available [26]. This study evaluated five pulse oximeters which were available on the market. Device performance in all five pulse oximeters in relation to agreement with a reference standard varied largely and did not reflect the differences seen when tested in a controlled laboratory environment against a simulator. This shows the importance of field-testing devices in the real-life settings and not only relying on performance results from simulators or laboratory testing alone. The reasons for these differences reflect the complexities of introducing new technologies in low resource settings, including training health workers on their consistent and correct use. The need for an adequate development and evaluation process for diagnostic aids in low resource settings has also been highlighted recently in the literature [27].

Most health workers (CHWs and FLHFWs) were able to use the devices to record a SpO_2_ measurement and the failure rate after three attempts was just 4·5%. Handheld devices had a lower failure rate (1–2%) than the fingertip devices (4–6%). The Masimo mobile phone pulse oximeter had the best overall performance across both primary and secondary outcome measures and in both age strata. This may be due to the motion signal processing techniques incorporated in the Masimo pulse oximeters to reduce motion artefact [28], which is important when using these devices on young children. These types of mobile phone pulse oximeters are a new emerging technology and should be further evaluated for these settings [11,29], especially given the current focus on multimodal and mHealth decision support platforms such as MEDSINC and Feebris [30,31].

While a high level of variability was seen in device performance, handheld pulse oximeters with multiple probes had higher agreement with the reference standard than the fingertip pulse oximeters and show similar levels of performance as previous studies on similar devices, where they reported mean differences of between −0·6 to 1·1 in four handheld pulse oximeters [32]. This reflects the fact that handheld devices allowed more precise measurements through the use of both paediatric and neonatal probes. While the importance of probe fit has been highlighted in the literature in relation to safer surgery [11,33,34], it is also a vital consideration for the assessment of respiratory and other illnesses in children. Our data show that fingertip devices had poorer performance in the younger age group, likely due to poor probe fit. Similar challenges with probe fit in younger children has also been documented in a smaller study conducted in Malawi and Bangladesh [35].

The finger-tip devices were also found to consistently record lower readings compared to the reference standard in both user groups and age strata, implying that hypoxaemia would be over diagnosed, leading to unnecessary referrals to higher level health facilities if these devices were used as screening devices for severe illness in children. These results are consistent with those of another study done evaluating similar types of fingertip pulse oximeters [36], which documented inaccurate readings and large errors, and suggested that these devices not be used as diagnostic tools by frontline health workers for severe illness in low resource settings. Performance times were better for the fingertip pulse oximeters than for the handheld devices, likely because they did not require having to attach the correct probe before starting the measurement.

While most frontline health workers could use all devices, FLHFWs were found to perform better using pulse oximeters than CHWs. This could be due to FLHFWs being more educated or having more familiarity with medical devices of this type, which was also reported in a recent qualitative study in Bangladesh and Malawi [35]. However, a number of recent studies have highlighted the need for adequate training for all cadres of health workers to ensure the effective scale-up of pulse oximeters, as measurement is generally easy to do, but the interpretation of the result being more nuanced [37,38].

A limitation of this study was that severely sick children were excluded for safety, which resulted in a more limited spectrum of oxygen saturation levels in the study sample (i.e. less children with low SpO_2_ levels). To account for this, we conducted laboratory testing of the devices using simulators before the field evaluation to test their accuracy at a range of oxygen saturation levels, going as low as 80%. The reference standard for this study, the Masimo Root patient monitor and connectivity platform with Radical 7 pulse oximeter, was recommended by the study scientific advisory committee, due to its portability and suitability for field settings, and having been validated in paediatric and neonatal populations [39,40]. As one of the test devices was also a Masimo product, and hence calibrated in the same way as the reference standard, positive bias could have been introduced in the results and this should be considered when interpreting these results. Due to low patient load in the community or remote clinics, where the health workers in our study work routinely, the study was instead conducted in busy hospitals where the chances of enroling the required study sample was higher. As this is not the routine work environment for the majority of the health workers we cannot fully generalise the findings from our study to the routine work environment of the health workers. However, to mitigate for this bias, data on the usability, utility and acceptability of these test devices in the routine work setting of health workers was conducted in the four countries. These data will be presented elsewhere. All five pulse oximeters tested in this study performed consistently well when tested on simulators in the laboratory but had high variability in performance when tested by frontline health workers in the field. More expensive phone and handheld pulse oximeters with multiple age-specific probes perform better than less expensive fingertip devices. This finding should be considered when making procurement decisions in low resource settings. First level health facility workers using the five test devices had better agreement with the reference standard than community health workers. Again, this needs to be considered when evaluating which level of the health system would most benefit from the introduction of pulse oximetry at scale.

## Contributors

KB participated in the design of the study, supervised the study, participated in the data collection, analysis and interpretation, and drafted and wrote the manuscript. KK reviewed the medical literature, conceived and designed the study, supervised the study, participated in the data analysis and interpretation, drafted and reviewed the manuscript. MP designed the statistical analysis and analysed the data. AM AWS ED TH LM DN MO MP AS JN participated in the design of the study and participated in the data collection. MM designed and conducted the training. TA participated in the data interpretation, drafted and reviewed the manuscript. RI participated in the data interpretation. All co-authors reviewed and approved the final version of the manuscript.

## Data sharing statement

Data collected, including individual participant data and informed consent forms, can be made available where appropriate, to others by request from k.baker@malariaconsortium.org

## Funding

Bill & Melinda Gates Foundation [OPP1054367].

## Declaration of Competing Interest

All authors had full access to all the data in the study and accept responsibility to submit for publication. None of the authors has any conflict of interest to declare and all authors have completed the ICMJE form.

## Figures and Tables

**Table:** 

**No.**	**Attribute**	**Description**
1	Usability - ease of use	Easy for CHWs to use the device, i.e. can apply it appropriately e.g. attach the probe correctly, switch on the device, select the correct settings, complete the assessment to get a SpO_2_ result
2	High level of decision support	Allows the community health worker to detect the symptoms of severe illness without the need for decision making from them
3	Automation of diagnosis	Automatically provides the CHW with a diagnosis of severe illness
4	High accuracy of measured/calculated result	The pulse oximeter consistently provides an accurate measure of the SpO_2_
5	No or little literacy and numeracy required	The device only requires a very low level of literacy and/or numeracy to be operated by the CHW
6	No or little training required	The CHW only requires minimal amounts of training to be able to use the device effectively to detect the symptoms of severe illness
7	No or little familiarity with technology required	The CHW does not need any prior familiarity with technology to operate the device effectively to detect the symptoms of severe illness
8	Long operational life in the field – e.g. more than two years	The device (not probes) will have an operational life while being used by CHWs of more than 2 years
9	Does not require charging (solar, battery, grid)	The device does not require charging to be used by CHWs to detect the symptoms of severe illness
10	Does not require replaceable parts (non-rechargeable batteries, consumables)	The device does not require replaceable parts such as non-rechargeable batteries and/or consumables throughout its functional life in the field
11	Requires little or no maintenance	The device does not require any maintenance throughout its operational life when used by CHWs to effectively detect the symptoms of severe illness
12	High durability/mechanical robustness	The device will not break during normal use by the CHW in the detection of the symptoms of severe illness
13	High CHW confidence in measurements	The readings provided by the device support the CHW in relation to detecting the symptoms of severe illness
14	High caregiver acceptability of diagnosis	The readings provided by the device help and support the caregiver/parent in accepting the diagnosis offered by the CHW
15	High patient comfort	The device does not cause hurt or discomfort to the patient while being used by the CHW in the detection of the symptoms of severe illness
16	High portability	The device is easy to carry by the CHW during normal working
17	Easy to maintain hygiene	The device is hygienic and easy to maintain in this regard – i.e. doesn't require specialist cleaning procedures or products
18	Low price (less than $50)	The annualized device cost is less than $50 (Device = total package of device plus consumables such as batteries/probes and chargers)
19	High level of safety	The device provides a high level of safety when it is being used for the detection of the symptoms of severe illness
20.	Multi-functional (includes a minimum of RR and Pulse oximeter)	The device incorporates several applications for the detection and classification of the symptoms of severe illness
